# An Integrative Multi-Source Evidence Framework for Prioritizing Virulence-Associated Pathways in *Metarhizium brunneum*

**DOI:** 10.3390/genes16111363

**Published:** 2025-11-10

**Authors:** Jingyi Wen, Wei Wei, Jing Li, Hua Bai, Narisu, Rui Wang

**Affiliations:** 1 College of Veterinary Medicine, Inner Mongolia Agricultural University, Hohhot 010018, China; 2 Department of Animal Husbandry and Veterinary Medicine, Ulanqab Vocational College, Ulanqab 012000, China; 3 Key Laboratory of Clinical Diagnosis and Treatment of Animal Diseases, Ministry of Agriculture, National Animal Medicine Experimental Teaching Center, Hohhot 010018, China; 4 Keerzuoyi Middle Banner Livestock Workstation, Tongliao 029300, China

**Keywords:** entomopathogenic fungi, *Metarhizium brunneum*, pathway, PHI-base, MAPK signaling pathway, pathway scoring framework

## Abstract

Background: The entomopathogenic fungus *Metarhizium brunneum* (*M. brunneum*) is an effective biocontrol agent against various vector arthropods such as ticks, mosquitoes, and flies. However, its virulence mechanisms remain poorly understood, which hinders its broader application. This study aims to establish an integrative framework for prioritizing virulence-related pathways in *M. brunneum* to aid in the development of more effective biocontrol strategies. Methods: A multidimensional virulence pathway scoring framework was developed using publicly available protein annotation data of *M. brunneum*. This approach integrates protein pathway enrichment, Gene Ontology (GO) functional analysis, PHI-base virulence factor mapping, and literature-derived evidence. A total of 20 pathways were evaluated, and a scoring system was applied based on protein coverage, Gene Ontology Biological Process (GO-BP) support, PHI-base hits, and literature support. Results: Among the 20 pathways evaluated, five pathways, including MAPK signaling, apoptosis, endocytosis, carbon metabolism, and biosynthesis of secondary metabolites received the highest priority scores. These pathways were identified as key virulence-related candidates, supported by both functional annotation and existing biological evidence. Conclusions: The proposed framework provides a reliable and scalable strategy for prioritizing virulence pathways in entomopathogenic fungi. It offers a solid foundation for subsequent transcriptomic validation, target screening, and functional characterization. This framework can also be applied to other fungi, contributing to the development of optimized biocontrol formulations.

## 1. Introduction

Vector-borne arthropods such as ticks, flies, and mosquitoes serve as the primary transmitters of numerous zoonotic diseases, including dengue fever, Lyme disease, and Japanese encephalitis [1,2,3,4]. The emergence of resistance and ecological risks associated with traditional chemical control methods has driven increasing interest in sustainable biological alternatives [5]. The entomopathogenic fungus *M. brunneum* has shown great potential in this regard due to its broad-spectrum virulence and environmental compatibility, and has been demonstrated to effectively infect and kill various vector insects [6,7].

However, biocontrol formulations based on *M. brunneum* still suffer from limitations in practical applications, particularly their relatively slow killing speed compared to chemical insecticides [8]. This drawback is partly attributed to the fact that the virulence mechanisms of *M. brunneum* have not yet been systematically elucidated, hindering the optimization of formulation strategies to enhance pathogenic efficiency. Although numerous studies have investigated *M. brunneum*-based biocontrol agents, most have focused on individual virulence-related genes or factors, while systematic strategies for virulence pathway identification and prioritization are lacking. Furthermore, although functional annotation methods such as KEGG and GO enrichment analyses are widely used [9,10,11], they alone are insufficient to capture the pathogenic relevance of biological pathways, especially in the absence of experimental validation or functional prioritization frameworks. Understanding these virulence pathways can directly guide the design of more effective biocontrol formulations by enhancing pathogenic efficiency, improving host invasion, and increasing resistance to environmental stress. We hypothesize that the integration of multisource functional evidence, including protein annotation, KEGG/GO enrichment, PHI-base mapping, and literature support, can effectively prioritize virulence pathways in *M. brunneum*, providing a more reliable framework for biocontrol formulation development.

Therefore, there is a need for an integrative framework to evaluate and prioritize virulence-associated pathways at the systems level. In this study, a multidimensional virulence pathway scoring framework was established based on publicly available protein annotation data of *M. brunneum*. By integrating protein-level pathway enrichment, GO functional association, PHI-base virulence factor mapping, and literature-derived evidence, candidate virulence pathways were prioritized. This approach not only provides a basis for subsequent transcriptomic verification, target screening, and functional characterization, but also offers a reproducible strategy for pathway-level virulence exploration in other entomopathogenic fungi.

## 2. Materials and Methods

### 2.1. Data Source

The genomic architecture of the genus *Metarhizium* is highly conserved [12]. To maximize the utilization of available genomic resources, the publicly available protein sequence dataset of the reference strain *M. brunneum* ARSEF 4556 (NCBI RefSeq assembly accession: GCF_013426205.1, file type: .faa) was retrieved from the NCBI database on 26 May 2025 and used as the basis for subsequent protein annotation and pathway analysis (https://www.ncbi.nlm.nih.gov/datasets/genome/GCF_013426205.1/, accessed on 26 May 2025).

### 2.2. Protein Annotation and Completeness Assessment

Functional annotation of the predicted protein sequences was performed using eggNOG-mapper v2, which assigns standardized biological classifications such as KEGG Orthologs (KO) and GO terms based on orthology inference [13]. These annotations were used for subsequent pathway identification and functional enrichment analyses.

To evaluate the completeness of the annotated protein set, BUSCO v5.8.0 was employed using the eukaryota_odb10 lineage dataset (creation date: 8 January 2024; *n* = 255) in “proteins” mode based on the predicted proteome [14]. A completeness cutoff of 95% was applied, which is considered the standard for high-quality genome annotation.

### 2.3. KEGG and GO Enrichment Analysis

KEGG pathway and GO-BP enrichment analyses were performed in “R version 4.4.1 (2024-06-14)” using all successfully annotated protein sequences as the background set. The top 20 KEGG pathways and the most frequent GO-BP terms were identified based on the number of annotated proteins mapped to each category. Statistical analysis and visualizations were conducted in R, and the enrichment results were displayed as bar plots.

### 2.4. Construction of a Virulence-Related Pathway Scoring System

A four-dimensional scoring framework was established to prioritize candidate virulence-related pathways, integrating protein-level evidence, GO-BP support, PHI-base hits, and literature support. Among these dimensions, PHI-base mapping and literature evidence directly reflect experimental support for pathway involvement in virulence and were therefore assigned the highest weight (0~3 points). In contrast, the number of annotated proteins and GO-BP functional support represent indirect indicators of pathway participation and do not provide functional validation; thus, these dimensions were assigned a lower weight (0~2 points) to balance the scoring system and prevent bias toward annotation abundance.

Although these weight assignments are empirical, they were designed to balance biological relevance and practical discrimination among pathways. This proportional scheme ensures that experimental evidence receives stronger emphasis while maintaining internal consistency across scoring dimensions.

Based on this multidimensional scoring framework, all annotated pathways in *M. brunneum* were systematically evaluated and ranked, resulting in the identification of high-priority pathways potentially associated with virulence. The final ranking matrix was visualized as a heatmap to illustrate comparative evidence support across pathways.

#### 2.4.1. Protein Coverage Scoring (0~2 Points)

Protein coverage was calculated to assess the degree of pathway representation at the protein annotation level. Based on eggNOG-mapper annotation results, the number of annotated proteins mapped to each candidate pathway (Gene Count) was compared with the total number of KEGG Ortholog entries defined for that pathway (KO Total) in the KEGG database. Coverage was computed as:

$$Protein\;coverage=\frac{Gene\;Count}{KO\;Total}$$

This metric reflects the annotation completeness and potential involvement of each pathway in *M. brunneum*. The rationale follows the concept of pathway completeness widely applied in metabolic network reconstruction and microbiome annotation [9,15,16].

**Figure 1:**
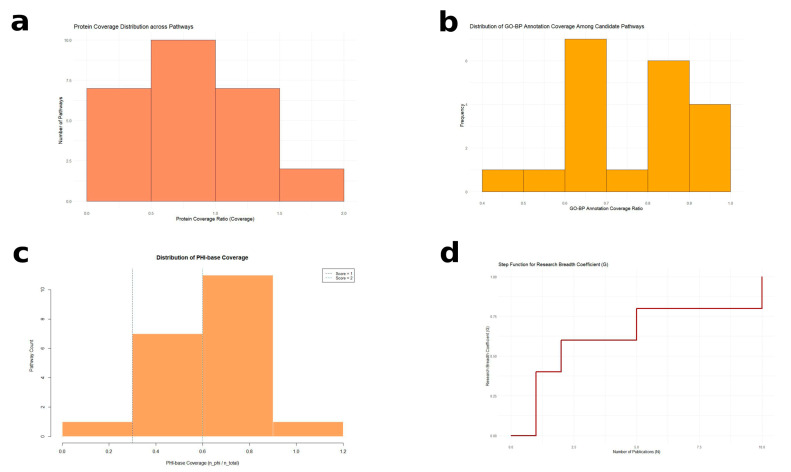
Distribution of scoring metrics for pathway prioritization. (**a**) Protein coverage distribution across candidate pathways. Most pathways showed protein coverage values between 0.5 and 1.5, exhibiting a right-skewed distribution. Based on this observation, a coverage threshold of ≥0.5 was defined as strong annotation support (score = 2), 0.2~0.5 as moderate support (score = 1), and <0.2 as weak support (score = 0), evaluating participation strength while reducing bias from gene family expansion. (**b**) Distribution of GO-BP annotation coverage. The majority of pathways displayed BP coverage in the range of 0.6~0.9, supporting the use of a coverage threshold of 0.8 as a high functional annotation criterion (score = 2). (**c**) Distribution of PHI-base coverage among pathways. Dashed lines indicate the two scoring thresholds (0.3 and 0.6) used to categorize the enrichment of virulence-associated proteins mapped from PHI-base. (**d**) Step function of the research breadth coefficient (*G*) based on the number of publications (*N*). The coefficient increases stepwise with *N*, reflecting the increasing breadth of literature evidence supporting each pathway.

According to the distribution of coverage values in this study (Figure 1a), pathways with Coverage ≥ 0.5 were defined as strongly supported by protein evidence. The scoring criteria were defined as follows: Coverage ≥ 0.5 was assigned 2 points, 0.2 ≤ Coverage < 0.5 was assigned 1 point, and Coverage < 0.2 received 0 points. To avoid overestimation caused by protein family expansion or functional redundancy, this metric was treated as a supporting indicator and integrated with other scoring dimensions in the overall pathway prioritization system.

#### 2.4.2. GO Functional Support Scoring (0~2 Points)

GO annotations for candidate pathway proteins were obtained from the eggNOG-mapper results. Only GO terms classified under the BP category were considered in this scoring dimension, as BP terms reflect functional involvement in cellular and biochemical processes [17]. For each pathway, the number of proteins annotated with BP terms (*n_bp_*) and the total number of proteins mapped to that pathway (*n_total_*) were calculated. BP annotation coverage was defined as:

$$BP\;coverage=\frac{n_{bp}}{n_{total}}$$

This metric reflects the functional annotation completeness of pathway-associated proteins and indicates their level of biological process involvement.

Based on the distribution of BP coverage across pathways (Figure 1b), the following scoring criteria were applied: BP coverage ≥ 0.8 was assigned 2 points, 0.4 ≤ BP coverage < 0.8 received 1 point, and BP coverage < 0.4 received 0 points. This metric supports the prioritization of pathways enriched with proteins that have well-defined biological roles, thus reflecting functional complexity and potential relevance to virulence.

#### 2.4.3. PHI-Base Hit Scoring (0~3 Points)

To assess virulence relevance of each candidate pathway, BLASTp searches were performed against PHI-base using TBtools v2.371 to identify proteins associated with experimentally verified virulence phenotypes [18,19]. For each pathway, the number of proteins with PHI-base matches (*n_phi_*) and the total number of annotated proteins in the pathway (*n_total_*) were determined. Virulence coverage was calculated as:

$$Virulence\;coverage=\frac{n_{phi}}{n_{total}}$$

Scoring was assigned based on the enrichment of virulence-associated proteins within each pathway. Pathways with virulence coverage ≥ 0.6 were assigned 3 points, 0.3 ≤ coverage < 0.6 received 2 points, and 0 < coverage < 0.3 received 1 point; pathways with no PHI-base hits received 0 points. These thresholds were established based on the empirical distribution of PHI-base hit ratios in this study (Figure 1c). This scoring dimension reflects the degree to which each pathway is enriched in known virulence-related genes and provides direct evidence of virulence potential.

#### 2.4.4. Literature Support Scoring (0~3 Points)

A literature-based scoring strategy was used to evaluate prior experimental evidence linking each pathway to fungal virulence. Publications were retrieved from the PubMed database (updated on 2 July 2025) using pathway names combined with the keywords “entomopathogenic fungi”, “*Metarhizium*”, “*Beauveria*”, “virulence”, “pathogenicity”, and “infection”. For signaling pathways, pathway component genes (e.g., “MAPK”, “Hog1”, “Fus3”, “Slt2”) were included to improve retrieval sensitivity. Host-centered studies, review articles, and publications lacking mechanistic relevance were excluded.

We focused on studies published before 2 July 2025, with no language restrictions. Excluded were articles without mechanistic relevance, review papers, or those centered on the host. A total of 55 articles were initially screened for relevance based on keywords. After applying inclusion and exclusion criteria, such as experimental validation and relevance to the study objectives, a subset of articles was selected for detailed analysis and scoring.

Each publication was assigned an evidence score according to Table 1 (0~3 points). For each pathway, the mean evidence score was calculated as:

$$Mean\;evidence\;score=\frac{1}{N}\sum_{i=1}^{N}s_{i}$$

where *s_i_* is the evidence score of publication *i*, and *N* is the number of supporting publications. A research breadth coefficient (*G*) was applied to adjust for variability in literature volume (Figure 1d):

$$G=\left\{\begin{array}{ll} 1.0, & N\geq 10\\ 0.8, & 5\leq N<10\\ 0.6, & 2\leq N<5\\ 0.4, & N=1\\ 0, & N=0 \end{array}\right.$$

The literature support score for each pathway was calculated as:

Literature support score=Mean evidence score×G

This metric reflects both the strength and breadth of existing experimental evidence and provides comparative literature-based support among pathways.

## 3. Results

### 3.1. BUSCO Completeness Assessment and Protein Annotation Overview

The BUSCO analysis showed that the annotated protein set of *M. brunneum* exhibited high completeness, with 98.8% completeness (250 single-copy and 2 duplicated BUSCOs), 0.8% fragmented, and 0.4% missing out of 255 benchmark ortholog groups. These results indicate that the genome annotation is of high quality and provides a reliable foundation for downstream functional analyses.

A total of 10,517 protein sequences were functionally annotated using eggNOG-mapper, among which 4389 proteins were assigned KO identifiers (KO coverage: 41.73%) and 3902 proteins received GO annotations. These annotations provided essential functional information for pathway enrichment analysis and the prioritization of virulence-related pathways in subsequent analyses. These results ensured sufficient annotation depth for subsequent pathway enrichment and virulence prioritization analyses.

### 3.2. Functional Annotation Overview of the M. brunneum Proteome (KEGG and GO)

**Figure 2:**
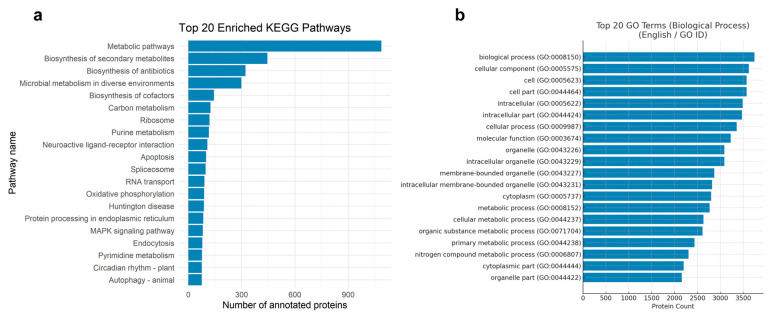
Functional annotation overview of the *M. brunneum* proteome. (**a**) Top 20 KEGG pathways enriched by annotated proteins. (**b**) Top 20 GO-BP terms associated with the annotated proteins.

The top 20 KEGG pathways, ranked by the number of annotated proteins, are shown in Figure 2a. Proteins were predominantly enriched in general metabolic pathways, including metabolic pathways, biosynthesis of secondary metabolites, and biosynthesis of antibiotics, indicating active primary and secondary metabolism in *M. brunneum*. Notably, several pathways potentially related to fungal virulence were also identified, including the MAPK signaling pathway, autophagy, and protein processing in the endoplasmic reticulum. These findings provided a preliminary functional landscape of the proteome and formed the basis for subsequent pathway prioritization and virulence relevance evaluation.

To further characterize the functional composition of *M. brunneum* proteins, GO classification was performed using eggNOG-mapper. Among GO-BP terms, the most frequently annotated categories were metabolic process, cellular process, and cellular component organization (Figure 2b). These results indicate that a large proportion of proteins in *M. brunneum* are involved in essential cellular activities, consistent with its complex metabolism and environmental adaptability.

### 3.3. Functional Evidence-Based Prioritization of Candidate Pathways

To evaluate the functional relevance of each candidate pathway to fungal virulence, we established a four-dimensional evidence scoring system incorporating protein coverage, GO-BP annotation support, PHI-base virulence gene hits, and literature-based validation.

**Figure 3:**
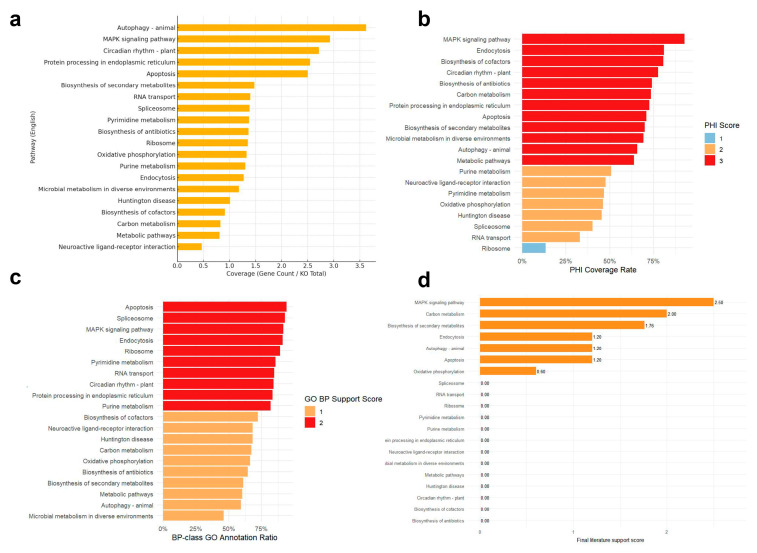
Multidimensional evidence framework used for pathway prioritization in *M. brunneum* (**a**) Protein coverage across the top 20 KEGG pathways, showing the gene count normalized by total KO. Higher protein coverage indicates more genes associated with the pathway, which reflects the overall representation of the pathway in the genome. (**b**) PHI-base virulence gene coverage ratio by pathway, with color representing the PHI score (blue: low coverage, red: high coverage). The PHI score indicates the extent to which each pathway is associated with known virulence factors in the PHI-base database. (**c**) GO-BP annotation support across pathways, displaying the proportion of genes annotated with relevant GO-BP. The ratio indicates the degree of functional annotation support for each pathway. (**d**) Literature-based evidence support, with scores representing the final literature support for each pathway.

Across the top 20 KEGG pathways identified in *M. brunneum*, protein coverage analysis revealed considerable variation in the proportion of annotated proteins per pathway (Figure 3a). Several pathways showed relatively high coverage, such as autophagy, MAPK signaling, and protein processing in the endoplasmic reticulum, indicating strong pathway representation at the proteome level.

PHI-base analysis further highlighted pathways enriched in known virulence-associated genes (Figure 3b). Pathways such as MAPK signaling, endocytosis, and biosynthesis of cofactors displayed the highest virulence gene coverage, suggesting their potential involvement in fungal pathogenicity. Conversely, multiple core metabolic pathways exhibited low PHI-base support, implying limited direct association with virulence.

GO-BP annotation analysis demonstrated that a subset of pathways was functionally enriched in biological processes relevant to stress adaptation, cellular regulation, and host interaction (Figure 3c). Notably, pathways such as apoptosis, spliceosome, and MAPK signaling showed strong functional coherence, reflected by high proportions of proteins annotated with BP terms.

Literature-based scoring provided an additional layer of biological validation by integrating experimental evidence from peer-reviewed studies (Figure 3d and Table 2). Among all pathways, the MAPK signaling pathway received the highest literature support score (2.50), followed by carbon metabolism (2.00), biosynthesis of secondary metabolites (1.76), and autophagy (1.20), all of which have been implicated in fungal virulence regulation across entomopathogenic and phytopathogenic systems.

Together, these multidimensional scoring results enabled the systematic prioritization of virulence-related pathways, providing a focused set of high-confidence candidates for downstream transcriptomic integration and functional validation.

**Figure 4:**
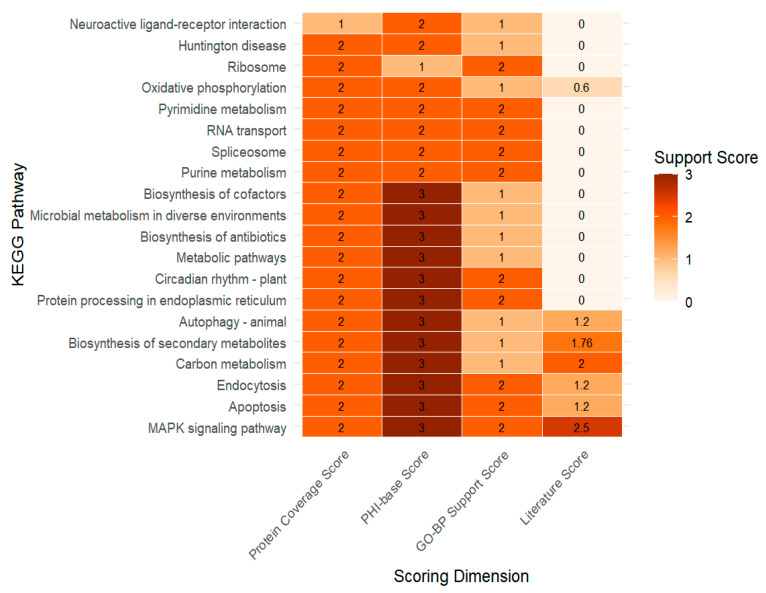
Integrated functional support score heatmap. The heatmap integrates scores across different evidence dimensions: Protein Coverage Score, PHI-base Score, GO-BP Support Score, and Literature Score. Each cell shows the aggregated support score for each pathway (3 = highest support, 0 = lowest support), with darker colors indicating higher support across dimensions.

To comprehensively evaluate the contribution of each evidence dimension, an integrated heatmap was constructed based on the four scoring metrics (protein coverage, PHI-base support, GO-BP annotation, and literature evidence) (Figure 4). The heatmap revealed substantial heterogeneity among pathways, with only a subset showing strong and consistent support across multiple evidence layers. In particular, the MAPK signaling pathway, apoptosis, endocytosis, carbon metabolism and biosynthesis of secondary metabolites exhibited the highest cumulative support and were ranked as top candidate virulence-associated pathways. These pathways showed strong PHI-base enrichment and functional relevance, supported by literature evidence implicating them in fungal pathogenicity, stress adaptation, and host interaction. Therefore, they were selected as high-priority pathways for downstream transcriptomic validation and mechanistic investigation.

## 4. Discussion

### 4.1. Biological Significance of the Top Five Pathways in Fungal Virulence

The top five pathways identified in this study play essential roles in fungal virulence mechanisms. The MAPK signaling pathway is crucial for regulating fungal virulence mechanisms. It controls key virulence traits such as conidiation, penetration ability, stress sensitivity, and host adaptation. By modulating transcription factors and kinases, MAPK signaling enables fungi to respond to environmental changes and host immune defenses, enhancing pathogenicity [20,21,22,23,24,25,26,27,28,29,30,31,32,33,34,35,36,37,38,39,40,41,42]. 

Apoptosis and endocytosis are also important processes linked to fungal virulence. Apoptosis regulates spore production, stress tolerance and fungal adaptation inside the host. By maintaining the balance between cell survival and death, apoptosis influences both fungal fitness and virulence [42,43,44]. Meanwhile, endocytosis (e.g., peroxidase-related activity) contributes to virulence expression. Dysregulation of endocytic function may reduce internalization capacity, thereby affecting fungal colonization and virulence [45,46]. Together, apoptosis and endocytosis participate in fungus–host interactions and help fungi survive immune pressure and enhance infection capability.

Carbon metabolism and secondary metabolite biosynthesis are directly related to fungal metabolic regulation. Downregulation of carbon metabolism has been shown to reduce fungal virulence by affecting toxin biosynthesis, penetration capacity, hyphal differentiation and metabolite production [47,48,49,50,51,52,53,54]. Secondary metabolite biosynthesis, especially the production of fungal toxins and bioactive compounds, is also a key determinant of virulence [50,53,55,56,57]. Carbon metabolism and secondary metabolism are functionally interconnected and jointly regulate fungal energy balance and pathogenicity.

In summary, these pathways complement each other in fungal virulence mechanisms. By regulating biological processes such as stress response, metabolic homeostasis and immune evasion, they collectively contribute to fungal pathogenicity.

### 4.2. Applicability and Limitations of the Multidimensional Pathway Scoring Framework

In studies of pathogenic mechanisms of entomopathogenic fungi, there is currently no unified system for prioritizing virulence-related pathways. Traditional approaches usually rely on KEGG or GO enrichment results for pathway screening, but these methods mainly reflect functional annotation and metabolic participation. They do not effectively distinguish between essential metabolic pathways and pathways that play central roles in virulence regulation. As a result, research directions may become scattered or biased during early-stage studies. To address this limitation, it is necessary to introduce a pathway prioritization strategy that integrates multiple sources of functional evidence prior to experimental verification, thereby improving the specificity and efficiency of virulence mechanism exploration.

The four-dimensional scoring framework established in this study—combining literature support, GO-BP functional annotation, protein coverage, and PHI-base virulence gene mapping—provides a multi-level evaluation strategy for identifying high-confidence virulence-associated pathways. This framework integrates prior biological knowledge with functional annotation, overcoming the limitations of single-dimension enrichment analysis and enabling a more interpretable and quantitative prioritization of pathways. However, this approach also has limitations. That the scoring system depends heavily on the available entries in the PHI-base database and the extent of literature coverage. This reliance may lead to potential bias, particularly against pathways that have been under-studied or are less represented in the PHI-base or literature. As a result, such pathways may receive lower scores, potentially underestimating their relevance in fungal virulence. Second, genetic variation among fungal strains may lead to differences in virulence-related genes. Since this study is based on the reference strain *M. brunneum* ARSEF 4556, the applicability of the scoring framework to other isolates requires further validation.

Overall, this scoring system is more suitable as a preliminary screening tool for fungal virulence pathway analysis rather than a definitive conclusion. To further validate the predictive value of our model, future studies are planned to incorporate dynamic multi-omics data, such as transcriptomic profiling at multiple infection stages, proteomics, and metabolomics, combined with functional genetics approaches. These include gene knockout experiments or chemical inhibition of candidate pathways to assess their impact on virulence phenotypes.

Furthermore, the framework can be extended to comparative analyses of virulence-related pathways in other entomopathogenic fungi, such as *Metarhizium anisopliae*, *Beauveria bassiana*. This extension would help highlight shared pathogenicity mechanisms, offering a deeper understanding of the evolution of virulence factors across fungal species and potentially identifying cross-species targets for biocontrol. Such comparative studies could ultimately inform the development of more efficient and tailored biocontrol strategies.

This strategy will help refine prioritization results, enhance biological interpretability, and ultimately guide biocontrol formulation. Our integrative approach thus offers a practical and expandable reference framework for virulence pathway screening, especially in early-stage studies or resource-limited research settings.

## 5. Conclusions

In this study, protein functional re-annotation and KEGG/GO enrichment analysis were performed based on the reference strain *M. brunneum* ARSEF 4556, and multiple candidate virulence-related pathways were systematically identified. By integrating protein coverage, PHI-base virulence gene support, GO-BP annotation and literature evidence, we developed a multidimensional pathway scoring framework to evaluate the functional relevance and prioritization of virulence-associated pathways. This framework provides a structured and evidence-based approach for pathway selection prior to experimental validation and offers a theoretical basis for dissecting the molecular mechanisms of fungal pathogenicity.

Through this evaluation system, five pathways—MAPK signaling, apoptosis, endocytosis, carbon metabolism and biosynthesis of secondary metabolites—were identified as high-confidence virulence-related candidates. These pathways are supported by functional annotation and previous biological evidence, and thus represent key directions for subsequent transcriptomic validation and functional genetic analysis. The framework established in this study is particularly applicable to early-stage mechanism studies or research scenarios with limited experimental resources.

Overall, this study proposes a reproducible strategy for virulence pathway prioritization in entomopathogenic fungi. Future research integrating multi-omics data and experimental validation will further refine this framework and enhance its applicability in elucidating the molecular basis of fungal infection. By combining these approaches, we aim to further validate the predicted pathways and improve the robustness of our model. This strategy will provide insights into shared pathogenicity mechanisms and inform biocontrol strategies.

## Figures and Tables

**Table 1:** Scoring criteria for literature-based evidence supporting pathway virulence relevance.

Score	Evidence Level	Evidence Description
3	Strong support	Functional validation (gene knockout/overexpression affects virulence)
2	Moderate support	Mechanistic association with virulence suggested by experimental data
1	Weak support	Expression changes only; indirect evidence
0	No support	No evidence of virulence relevance

Literature evidence was retrieved from PubMed (search updated to 2 July 2025) using pathway-specific queries combined with virulence-related terms. Review articles and host-centered studies were excluded.

**Table 2:** Literature-based evidence scores for candidate KEGG pathways in *M. brunneum*.

Pathway	No. of Studies (N)	Mean Evidence Score (Avg)	Breadth Coefficient (G)	Final Literature Support Score
Metabolic pathways	0	0	0	0
Biosynthesis of secondary metabolites	5	2.2	0.8	1.76
Biosynthesis of antibiotics	0	0	0	0
Microbial metabolism in diverse environments	0	0	0	0
Biosynthesis of cofactors	0	0	0	0
Carbon metabolism	10	2	1.0	2.0
Ribosome	0	0	0	0
Purine metabolism	0	0	0	0
Neuroactive ligand-receptor interaction	0	0	0	0
Apoptosis	3	2	0.6	1.2
Spliceosome	0	0	0	0
RNA transport	0	0	0	0
Oxidative phosphorylation	2	1	0.6	0.6
Huntington disease	0	0	0	0
Protein processing in endoplasmic reticulum	0	0	0	0
MAPK signaling pathway	26	2.5	1.0	2.5
Endocytosis	2	2	0.6	1.2
Pyrimidine metabolism	0	0	0	0
Circadian rhythm—plant	0	0	0	0
Autophagy—animal	1	3	0.4	1.2

To avoid scoring bias caused by pathway generalization, for literature that hits Metabolic pathways, if its content clearly supports a specific metabolic sub-pathway (such as carbon metabolism), its score will be attributed to the corresponding sub-pathway and its original hit pathway will be indicated in the record.

## Data Availability

The original contributions presented in this study are included in the article. Further inquiries can be directed to the corresponding author.

## Abbreviations

The following abbreviations are used in this manuscript:

*M. brunneum*	*Metarhizium brunneum*
GO	Gene Ontology
KO	KEGG Ortholog
BP	Biological Process
